# Cross-sectional survey to assess the status of antimicrobial stewardship programs in public Chilean hospitals

**DOI:** 10.1186/s13756-026-01703-0

**Published:** 2026-03-17

**Authors:** Ruth Rosales, Claudio González, José Valderrama, Tomás Reyes-Barros, Carmen Gloria Núñez, Báltica Cabieses, José M. Munita, Tania Herrera

**Affiliations:** 1 Multidisciplinary Initiative for Collaborative Research on Bacterial Resistance (MICROB-R), Ministerio de Salud, Santiago, Chile; 2 Hospital Barros Luco Trudeau, Gran Av. José Miguel Carrera 3204, San Miguel, Región Metropolitana Chile; 3 Departamento de Enfermedades Infecciosas del Adulto, Escuela de Medicina, Pontificia Universidad Católica de Chile, Santiago, Chile; 4 Red de Salud UC CHRISTUS, Santiago, Chile; 5 Instituto de Ciencias e Innovación en Medicina, Facultad de Medicina, Clínica Alemana, Universidad del Desarrollo, Santiago, Chile; 6 Surveillance, Epidemiology, and New Technologies for Infectious Emerging (SENTINET), Santiago, Chile; 7 Ministerio de Salud, Gobierno de Chile, Santiago, Chile

## Abstract

**Background:**

Antimicrobial resistance is a leading cause of death worldwide, with the highest burdens in low-resource settings. Antimicrobial stewardship programs (ASP) are coordinated interventions designed to improve and measure the appropriate use of antimicrobials. In December 2020, the Ministry of Health of Chile mandated that all hospitals in the country implement an ASP, but there has been little rigorous, comparable information on the prevalence and types of ASP activities currently occurring in Chilean hospitals, which is critical for infection control officials and health decision-makers.

**Objectives:**

We aimed to assess the current degree of implementation of ASPs in Chilean public hospitals using a web-based survey.

**Results:**

Of 66 responses, 57 hospitals (86.4%) reported having an ASP and were used as the denominator in the analyses. Among them, 39 (68.4%) reported having a written institutional protocol. On average, the hospitals performed seven antimicrobial optimization activities (range 1–9). For hospitals that have WHO Reserve group antibiotics, use authorization was almost universally required (90.9% to 100%). Prospective audit and feedback was performed in 64.9% of the institutions. Seventeen hospitals (29.8%) did not measure their antibiotic consumption. The median ASP implementation survey score was 68.4 points (ranging from 30.7 to 96.5).

**Conclusion:**

These findings show a variable implementation of ASP activities in Chilean hospitals and provide the necessary information to establish the baseline for the implementation of ASPs in hospitals in Chile.

**Supplementary Information:**

The online version contains supplementary material available at 10.1186/s13756-026-01703-0.

## Introduction

 Antimicrobial resistance (AMR) is a major healthcare problem worldwide, with a particularly high burden in low-resource settings [1]. Numerous professional and governmental organizations recommend antibiotic stewardship programs (ASPs) as part of the key strategies to tackle AMR [2–4]. ASPs foster the appropriate use of antimicrobials with the aim of improving clinical outcomes while minimizing toxicity and limiting the development of resistant organisms [5]. Several elements are deemed crucial for the successful establishment of ASPs, including an identifiable leader, direct dependence of hospital leadership, and dedicated resources and staff, among several others [6]. The most important of them are known as ‘core elements’ and have been clearly defined by the United States Centers for Disease Control and Prevention (CDC) and other international guidelines [7].

The capacity to implement ASP varies among countries. Low- and middle-income countries have fewer ASP capabilities than high-income countries, likely reflecting funding constraints, lack of infrastructure and limited political commitment [8]. Nevertheless, recent data suggest hospital ASP initiatives have increased over time across Latin America [9]. In 2017, Chile launched the first version of the National Plan against AMR, aimed at reducing the emergence and dissemination of AMR [10]. Among several actions, a nationwide mandate to implement ASPs across all medium and high-complexity hospitals was published in December 2020. This regulation established the minimum structure required for ASP teams, including an infectious diseases specialist, a clinical pharmacist, and a microbiology specialist. It also mandated the formal establishment of the team through an official resolution, the development of an operative plan and the monitoring of indicators requested by central authorities, while recommending specific strategies for implementation. In addition, a national coordinating body was defined, so that teams could seek guidance regarding implementation.

However, during this period—amid the COVID-19 pandemic—it became increasingly difficult for hospital teams to respond to this mandate. As a result, the mandate had to be reiterated on at least two additional occasions. Despite these challenges, the establishment of the regulation strengthened the work that ASP teams had already been carrying out for years in several hospitals across the country, and it also provided support for the conformation of new teams that were promoting this initiative at the local level [11]. At that time, data regarding the state of ASPs in Chilean hospitals were lacking. Therefore, in this study we set out to assess the degree of implementation of ASPs in Chilean hospitals using a nation-wide online survey.

## Methods

### Ethical considerations

This study was approved by the Institutional Review Board of the Southern Metropolitan Health Service in Santiago, Chile. Approval number 20-16-03-2020 in memorandum no 109/2020.

## Study setting

The Chilean healthcare system has a public and a private component [12]. The former serves around 80% of the population, including lower socio-economic groups in both urban and rural settings, as well as retirees [13]. The National Ministry of Health (MINSAL) categorizes healthcare centers in high-, medium-, and low-complexity, according to their technical capacities [14]. As mentioned, in December 2020, the General Technical Standard No. 210 for the rationalization of the use of antimicrobials in clinical care was officially approved [11], mandating all medium- and high-complexity hospitals in the country to implement an ASP within six months.

## Study design

This was a cross-sectional, quantitative study. Data were obtained from an online survey directly distributed by the Ministry of Health to all medium- and high-complexity healthcare centers across the Chilean territory.

## Data-collection instrument

A literature search was undertaken of published ASP standards and of previously applied surveys using Pubmed, SCIELO, Epistemonikos, and Google Scholar in 2019. Search terms included “Survey” AND (“Antimicrobial” OR “Antibiotic” OR “Stewardship” OR “Health Plan Implementation”). All articles published over the last 10 years in English or Spanish were evaluated. Manuscripts carried out in psychiatric, cosmetic surgery, plaster pavilions, maternity hospitals, and geriatric establishments were excluded. Three research manuscripts on ASP surveys in health centers [15–17] and three documents from scientific societies or government agencies providing guidance on ASP implementation were identified [3, 7, 18].

Based on these data, a draft survey was crafted including common core elements and previously published questions. A scoring system was distributed within the different categories of the survey as follows: structural organization (20%), human resources dedicated to ASP (20%), ASP activities (15%), implementation of treatment guidelines (10%), surveillance and monitoring of indicators (20%), and analysis and feedback of the information (15%). The scoring system implemented had a minimum of 0 and a maximum of 100 points. The limit value to establish compliance was arbitrarily set at 90 out of 100 points. An external committee of six epidemiology and ASP experts, including members of the Antimicrobials Committee of the Chilean Society of Infectious Diseases, reviewed the survey and scoring system. The evaluation included an in-person meeting with two authors serving as facilitators to incorporate suggestions and resolve differences (RR, TH). In February 2020, a final version of the consensus survey was obtained which was composed of 34 questions (Supplementary S1). Once finalized, the survey was transferred to a web-based format (SurveyMonkey^®^) to be directly distributed to all officials in charge of ASPs in medium- and high-complexity public hospitals, as per government records. The survey was distributed during March 2021 and May 2022. No economic incentives were provided to participants. In the absence of an official in charge, deputy medical directors were responsible for assigning a suitable person to complete the survey. Only one response was recorded per hospital.

## Analysis

Responses from all hospitals reporting to have an ASP in place were analyzed and assigned an overall score, along with individual scores for each of the survey’s sections. Antibiotics were classified in three groups as per the World Health Organization (WHO) AWaRe categorization: Access, Watch, and Reserve [19]. Variables were expressed as mean ± standard deviation (SD) or as median and interquartile range (IQR), as appropriate. Analyses were performed using RStudio 2022.12.0 and STATA 14.

## Results

Out of a total of 94 high- and medium-complexity healthcare centers identified in the country during the study period, 66 (70.2%) provided a response. Of them, 57 (86.4%) reported having an ASP in place and were used as the denominator in the analyses. Forty-two of those 57 (73.7%) were classified as high-complexity facilities. The median number of beds of centers with implemented ASPs was 289 (range 46–887), with 63% of them having between 101 and 500 and 17.5% with > 500 beds.

Among centers with an ASP in place, 39 (68.4%) reported having a written institutional ASP protocol and 38 (66.7%) had an official document detailing the activities and functions of the program, including formal leadership commitment. Only 30 (52.6%) of the programs had members of the clinical microbiology lab, infectious diseases, and clinical/hospital pharmacists as part of the program. Staff members with the highest presence in ASPs were clinical pharmacists and clinical microbiology professionals, while the lowest frequency obtained was for data analysts (Table 1).

**Table 1:** Description of ASP composition by professionals and weekly hours of dedication

	Type of personnel forming part of ASP, % (*n*)	Number of professionals per ASP, median (range)	Time spent per 500 beds (hours/week), median (range)
Clinical pharmacist	87.7 (50)	1 (0–7)	12 (0-198)
Clinical laboratory scientists	70.2 (40)	1 (0–3)	6 (0–98)
Infectious diseases specialist	64.9 (37)	1 (0–8)	17 (0-164)
Hospital Pharmacist	52.6 (30)	1 (0–3)	0 (0-164)
Nurse	43.9 (25)	0 (0–2)	4 (0–45)
Critical care specialist	38.6 (22)	0 (0–3)	0 (0–96)
Medical microbiologist	26.3 (15)	0 (0–3)	0 (0–65)
Administrative support	21.1 (12)	0 (0–1)	0 (0–38)
Data analyst	15.8 (9)	0 (0–1)	0 (0–9)

ASP, Antimicrobial stewardship

## ASP characteristics

**Fig. 1:**
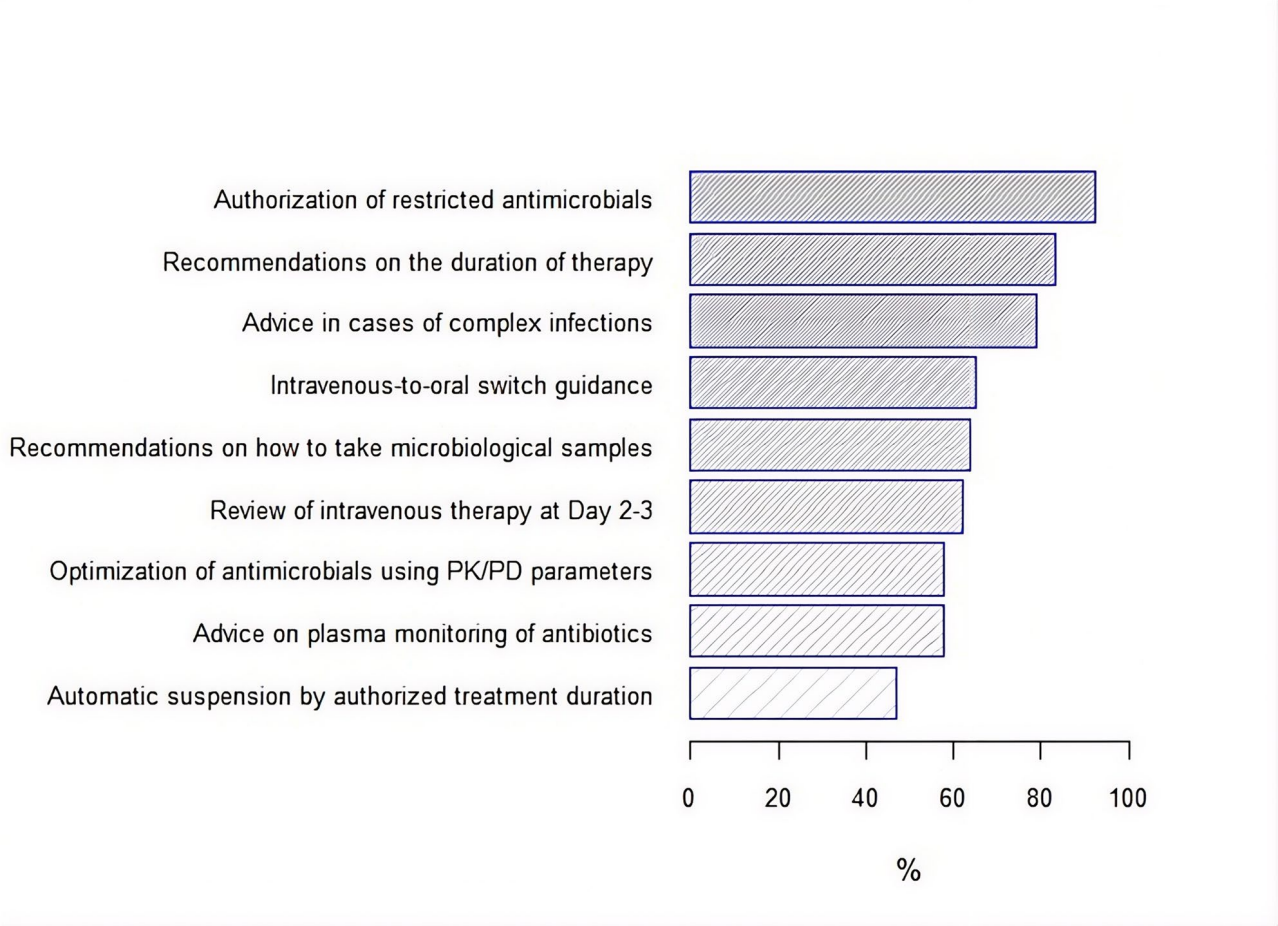
Overall prevalence of ASP activities in 57 hospitals

On average, ASPs declared to perform seven antimicrobial optimization activities on a regular basis, with a wide range among hospitals (1–9). The activities most frequently declared were “authorization of restricted antibiotics” and “recommendations on the duration of therapy” (Fig. 1), while 64.9% of programs reported performing “prospective audit and feedback”. Regarding the types of supervised clinical services, 80–90% of ASPs supervised antimicrobial use in critical care units and medical or surgical wards. Most hospitals (96.5%) referred to follow clinical guidelines to manage infectious diseases, of which 45.9% were locally-elaborated documents, and the rest corresponded to national or international standards. Surgical prophylaxis and urinary tract infections were the most frequently mentioned clinical guidelines (Supplementary, Table S1).

Various pharmacy-led activities were evaluated (Table 2). For WHO *Reserve* group antibiotics—such as ceftaroline, ceftazidime/avibactam, ceftolozane/tazobactam, fosfomycin (IV), and tigecycline —all hospitals (100%) required pre-authorization (Table 3). In contrast, for *Watch* group antibiotics, this requirement ranged widely—from as low as 4.4% for oral erythromycin to 82% for meropenem (Supplementary Table S2).

**Table 2:** Prevalence of pharmacy-driven activities related to ASP for all 57 hospitals

Activity types	% (*n*)
*Pharmacy activities*	
Use of a dedicated form for authorization of antimicrobial prescription	87.7 (50)
Deliver out-of-stock alerts	77.2 (44)
Established procedure to dispense antimicrobials along with the ASP team	71.9 (41)
Provide alerts to ASP regarding the initiation of restricted antimicrobials	66.7 (38)
Warns of medication errors related to antimicrobials	54.4 (31)
Existence of protocols to guarantee the safe administration of antimicrobials	38.6 (22)
Alerts the ASP team of prolonged antimicrobial treatments (> 10 days)	35.1 (20)
Delivers alerts for suspension of surgical prophylaxis when exceeding the prespecified time	17.5 (10)

ASP, Antimicrobial stewardship

**Table 3:** Prevalence of the requirement for preauthorization forms for antibiotics in the reserve group of the aware classification

Reserve class	Hospitals requiring pre-authorization % (*n*^a^)
Ceftaroline	100 (2/2)
Ceftazidime/avibactam	100 (22/22)
Ceftolozane/tazobactam	100 (6/6)
Fosfomycin (IV)	100 (2/2)
Tigecycline	100 (26/26)
Linezolid (PO)	96.8 (30/31)
Colistin	94.1 (32/34)
Linezolid (IV)	92.7 (38/41)
Aztreonam	90.9 (10/11)
Daptomycin	90.9 (10/11)

IV, Intravenous; PO, Per oral

^a^Only hospitals that reported having each antibiotic in their arsenal were considered

A total of 52.6% of respondents used defined daily doses (DDD)/100 bed-days to measure antimicrobial usage, while only 7 (12.3%) reported using “days of treatment” (DOT). Notably, almost a third of ASPs (29.8%) did not use any metric to measure antimicrobial consumption. Among the clinical microbiology laboratory’s activities, “availability of rapid techniques for identifying resistant microorganisms” was the least often performed (Table 4).

**Table 4:** Prevalence of microbiology activities related to ASP for all 57 hospitals

Activity types	% (*n*)
Laboratory reports of critical results (e.g. positive blood cultures)	93.0 (53)
Clinical microbiological report delivered within 48–72 h	87.7 (50)
Resistance reports performed following CLSI recommendations	87.7 (50)
Periodical report of the incidence of antimicrobial-resistant organisms (e.g. carbapenemase-producing Enterobacterales)	80.7 (46)
Stratified susceptibility reports	73.7 (42)
Availability of rapid techniques for identifying specific resistant microorganisms	61.4 (35)

## Evaluation of interventions and educational activities

Twelve (21.1%) of the 57 ASPs reported correlating antibiotic consumption with AMR data, and 28 (49.1%) evaluated the costs associated with antibiotic consumption. A total of 31 (54.4%) centers reported cumulative susceptibility reports, and 18 (31.6%) performed periodic reports (annual or biannual) of antimicrobial consumption. Only 5 (8.8%) hospitals reported assessing the quality of prescription (compliance with guidelines).

In terms of educational activities, 28 (49.1%) programs provided education in clinical meetings, and 19 (33.3%) reported having ASP material as a part of the institutional induction process. A total of 30 (52.6%) hospitals reported that prescribers received feedback regarding antibiotic prescriptions during the infectious diseases team’s clinical rounds.

### Score of ASP compliance

**Fig. 2:**
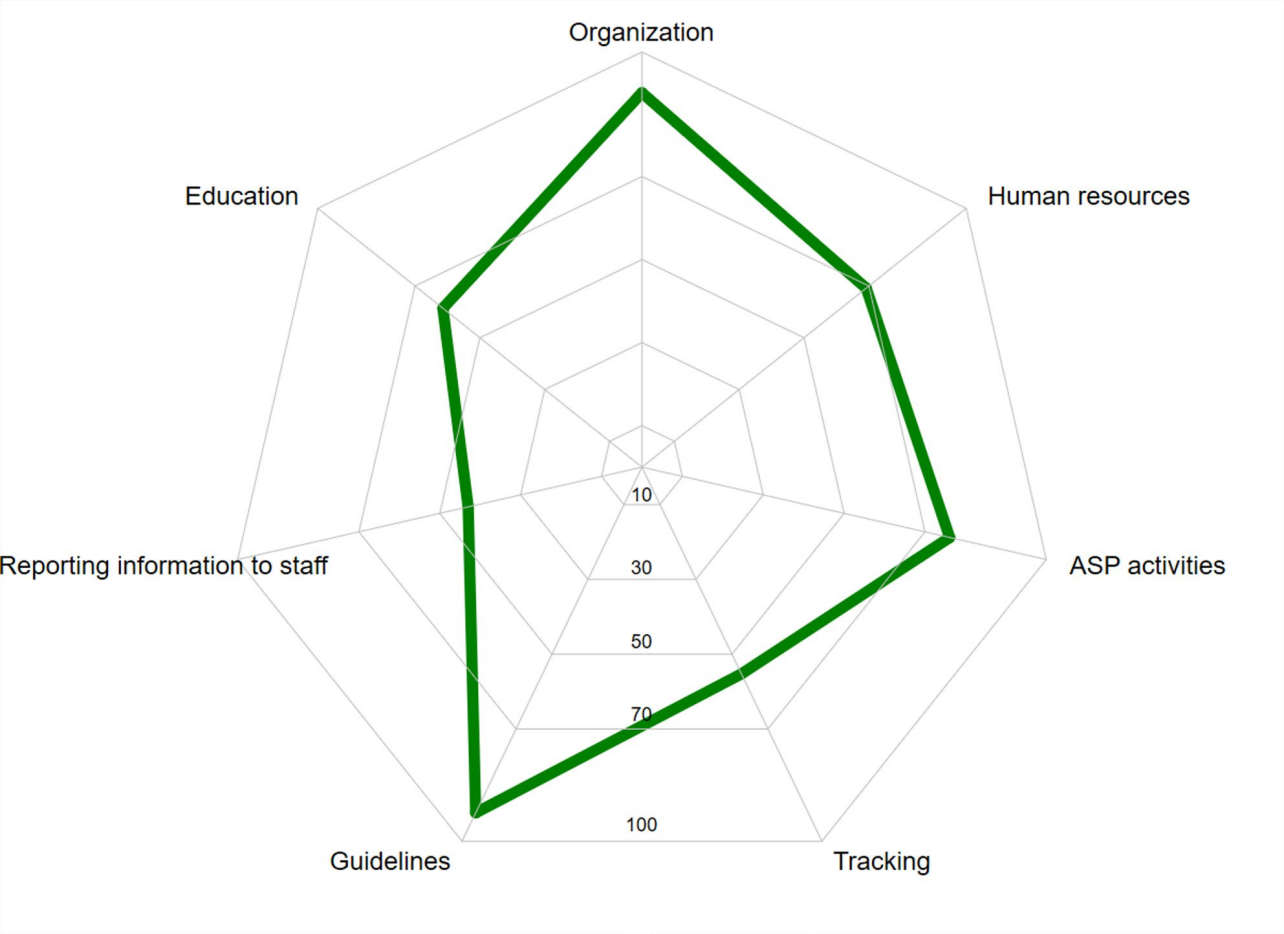
Mean overall performance by scoring item (0–100%)

The overall median score of ASPs was 68.4 points (range 30.7–96.5 points), with 49 of 57 (86%) reaching at least 50 points and only 4 (7%) scoring *≥* 90 points. High-complexity hospitals had a median score that was 17.9 points higher than medium-complexity hospitals (72.6 vs. 54.7, respectively; p < 0.001). Similarly, healthcare centers with > 500 beds also exhibited significantly higher scores than 101–500 beds (74.7 vs. 69.2, respectively) (Supplementary, Table S3). Figure 2 depicts the overall results for all evaluated categories. The lowest compliance was observed for the categories ”reporting information to staff’ and tracking”. The first relates to providing updates on ASP, disseminating information, and education to clinical personnel, while the latter refers to the formal evaluation of ASP and associated indicators.

## Discussion

We present the first nationwide assessment of the ASPs in Chile. Our data suggest that, after 15 months from the publication of a government standard mandating the creation of ASPs in all medium- and high-complexity hospitals, the level of implementation was far from ideal, with a minority of centers fulfilling internationally-recognized requirements to ensure a successful program. Indeed, while 57 of 66 respondents reported having an ASP in place, less than 10% of programs obtained > 90 points in our scoring system, which was largely based in fulfillment of the core elements. It should be noted, however, that both the regulation and the implementation of the instrument ocurred during the COVID-19 pandemic, a situation that placed pressure on hospitals and disrupted many health interventions.

Although 86% of the hospitals surveyed reported having an ASP team, about one-third of them did not have an institutional policy or a written ASP protocol. Moreover, although interventions in different areas of implementation were noted, important activities like estimating the correlation between antimicrobial consumption and bacterial susceptibility, or developing local empirical treatment guidelines, were infrequently performed, which suggests limited development of the governance structure. On the other hand, tracking and reporting, two core elements for the dissemination of the cumulative antibiogram and antimicrobial consumption data, has room for improvement since a high proportion of the centers did not report these data to clinicians. Finally, only five hospitals carried out activities that evaluate the impact of ASPs’ interventions, which is a crucial point in the development of an ASP.

The constitution of an ASP team that recognizes the different functions of its members is key for an effective ASP implementation [2]. Our data suggests that around 35% of programs do not have an infectious diseases specialist within the ASP team, a meagre figure compared to international surveys [20, 21]. Similar to Chile, specialist pharmacists and infectious diseases physicians are the primary ASP staff in North America [22] and Japan [23]. Pharmacists with advanced training in infectious diseases can play a critical role in the rational use of antimicrobials [24]. Our data indicate that, although the presence of these professionals in ASPs is relatively high, the number of hours they contribute per week remains limited. Nevertheless, it is notable that interventions aimed at individualizing antimicrobial therapy—practices commonly performed by pharmacists in developed countries and considered fundamental for enhancing treatment efficacy and safety, particularly in critically ill patients—were infrequently reported. It should be noted, though, that the applied survey did not assess the level of training of the ASPs’ clinical pharmacists, nor their specific expertise in antimicrobial stewardship. The limited involvement of pharmacists in ASP activities in our cohort likely stems from structural constraints, including insufficient real dedicated full-time equivalents (FTE) and protected time, understaffing, heavy operational workloads, limited leadership support, and a lack of expertise. A 2023 Latin American study documented these barriers and noted that hospital leadership often underestimates the role of pharmacists within ASP teams [25]. These findings can form the basis of a roadmap to strengthen the presence of this professional in ASP.

.

Clinical microbiologists are essential members of the ASPs, providing critical diagnostic data that inform targeted antimicrobial therapy and resistance surveillance [26]. However, their limited availability in Chile represents a significant barrier to the timely and effective implementation of key stewardship interventions.

Clear descriptions of the need for human resources to implement an ASP are scarce. However, there are some staff recommendations in the literature for ASP [27–29]. For example, the Dutch consensus report [27] suggests that an ASP team requires 1.25 to 3.18 FTE in personnel for the monitoring of at least three stewardship objectives, depending on the hospital’s size (calculated for hospitals between 300 and 1200 beds). Our data showed that, on average, ASP teams in Chile do not reach one FTE (44 h/week) per 500 beds, even when considering the effort of infectious disease specialists and clinical pharmacists combined. A relevant factor in this regard is that 66.7% of the hospitals had a policy establishing the functions of the ASP members, but the minimum required dedicated time remains unregulated, leaving space for policy improvement in the following years.

Our survey suggests some ASP practices have been widely adopted across different hospitals. For instance, prior authorization for restricted antimicrobial use was frequently reported, particularly among antimicrobials belonging to the Reserve WHO group. These results aligns with previously data reporting this intervention among the main ASP activities within low- and middle-income countries [7, 30]. Similarly, another frequently mentioned intervention was the recommendation of antimicrobial duration; however, it was seldom accompanied by automatic alerting systems, possibly due to a lack of electronic medical records and integrated prescriptions in most hospitals in Chile [31]. Clinical decision support systems have shown satisfactory results in different aspects of ASP activities [32], but resource limitations are likely hampering their use in developing regions.

One of the main strengths of this study was the adoption of a score to evaluate ASPs implementation locally. This score may be used to assess gaps and opportunities for improvement, but also to explore the impact of different aspects of ASPs’ implementation with clinical outcomes, as done previously [33]. In our study, none of the hospitals obtained the maximum score for compliance. Interestingly, we observed an association between the score obtained by hospitals and some of their characteristics such as complexity or number of beds. While this finding requires further exploration, it could reflect deficiencies in implementation caused by unequal institutional capacities between smaller and larger hospitals, especially given the regional differences observed in the country such as the distribution of human resources and the financial autonomy with which the different Health Services (health districts in Chile) operate [34].

Our study has several limitations. First, since only a subset of the 94 total hospitals provided a response, our results may be biased towards healthcare centers more likely to have an ASP in place, and therefore might be overestimating the status of such programs in the country. Nevertheless, with 70% of the hospitals answering the questionnaire, our data provide a representative landscape of the Chilean territory. Problems surveying hospitals have been previously detected in Latin American Hospitals [35] and highlight the need to couple government mandates with mandatory evaluations to ensure compliance [36]. Second, we only approached institutions from the public healthcare system. In Chile, the fragmentation of the healthcare system into private and public systems, has resulted in different management modalities [37]. While the national ASP norm mandates their implementation in all institutions, regardless of the financing types, lower levels of ASP implementation have been reported in non-public institutions in countries with similar healthcare systems [38]. Hence, the exclusion of private facilities could have overestimated the level of ASP implementation in Chile. Future studies should aim to approach all healthcare centers to improve the representation of different systems. Third, as with any survey-based study, questions might be subject to interpretation and respondents might have felt inclined to provide ‘appropriate’ answers, especially since the survey was distributed by the National Ministry of Health, which could bias the results. Ideally, future studies should include in-person visits and audits of participating institutions to strengthen the validity of their results. Fourth, our survey has methodological limitations: no formal piloting, no reliability or validity testing, and a public-sector-only sampling frame without non-response analysis or weighting. This may bias results toward more organized centers. Hence, the score is best viewed as a self-reported baseline, not a performance indicator. Future work should prospectively validate the survey by linking scores to objective outcomes, as demonstrated in other stewardship survey evaluations with solid methodologies [35, 39].

Despite these limitations, this study describes the first survey carried out by local experts on ASP in Chile and provides some important findings about the implementation of ASP in this country. These results can serve as a starting point for decision makers to continuously evaluate the progress in the implementation of ASPs at the local and national levels, especially considering that in the years following our survey significant progress was made in the formal establishment of ASP teams. Indeed, in 2024, official information from the Ministry of Health reports achieving 100% coverage of both the formal establishment of ASP teams and the development of a formal ASP work plan in medium- and high-complexity public hospitals [40]. This appears to have been primarily driven by the establishment of the ASP as a management commitment for the Health Services and hospitals, which provided a strong incentive for compliance with the regulation. Future work will focus on classifying hospital antimicrobial stewardship programs (ASPs) by maturity level (basic, intermediate, and advanced) and evaluating the effect of ASP implementation on hospital antimicrobial consumption. Future applications of our survey in hospitals, conducted annually at the central level, could be a national strategy for evaluating ASP.

## Conclusions

ASPs are a global strategy to preserve the proper use of antimicrobials in hospitals. In Chile, a country where the implementation of ASPs has become mandatory for medium- and high-complexity hospitals, at the moment when this survey was applied there were gaps in some important aspects of ASPs, especially regarding team formation. Even more, for hospitals that were already in the process of developing the program, there were even greater gaps in training and in the evaluation of the implemented strategies. Nevertheless, this study provides necessary information to establish a baseline in the implementation of ASPs in a significant number of hospitals in Chile and it also provides objective information that can be used to propose alternatives for improvement. Finally, the survey and the proposed score system could be a measurement tool for monitoring the individual progress of institutions.

## Supplementary Information

Below is the link to the electronic supplementary material.

Supplementary Material 1

## Data Availability

No datasets were generated or analysed during the current study.
