# Supplementary material for: Cross-sectional survey to assess the status of antimicrobial stewardship programs in public Chilean hospitals
Source: Antimicrob Resist Infect Control. 2026 Mar 17;15:38. doi: 10.1186/s13756-026-01703-0 (PMC12998113; doi:10.1186/s13756-026-01703-0)
Supplement: Supplementary file 1 — Supplementary Material 1 [file 13756_2026_1703_MOESM1_ESM.docx]

Type of the Paper (Article, Review, Communication, etc.)

Cross-sectional Survey to Assess the Status of Antimicrobial Stewardship Programs in Public Chilean Hospitals

**Ruth Rosales 1,2, Claudio González 1, José Valderrama1, Tomás Reyes-Barros 3 Carmen Gloria Núñez 1, Báltica Cabieses 2, José M. Munita 2,4, Tania Herrera5**

a. 1 Hospital Barros Luco Trudeau. Gran Av. José Miguel Carrera 3204, San Miguel, Región Metropolitana. Chile

2 Multidisciplinary Initiative for Collaborative Research On Bacterial Resistance (MICROB-R).

Ministerio de Salud, Chile

3 Departamento de Enfermedades Infecciosas del Adulto, Facultad de Medicina, Pontificia Universidad Católica de Chile.

4 Instituto de Ciencias e Innovación en Medicina, Facultad de Medicina Clínica Alemana - Universidad del Desarrollo, Chile.

5 Ministerio de Salud, Gobierno de Chile

* Correspondence: rurosalesch@gmail.com

**Table S1** : Presence of local implemented guidelines

| **Guidelines** | **%(n)** |
| --- | --- |
| Surgical prophylaxis | 71.9 (41) |
| Urinary tract infection | 47.4 (27) |
| Community-acquired pneumonia | 40.4 (23) |
| Clostridium difficile diarrhea | 36.8 (21) |
| Ventilator-associated pneumonia | 35.1 (20) |
| Bloodstream infection | 31.6 (18) |
| Febrile neutropenia | 31.6 (18) |
| Osteoarticular infections | 26.3 (15) |
| Skin and soft tissue infection | 24.6 (14) |
| Meningitis | 24.6 (14) |
| Methicillin-resistant S.aureus infection (MRSA) | 17.5 (10) |
| Influenza | 12.3 (7) |

Table S2. Prevalence of antimicrobial restriction belonging to institutional formularies according to AWARE class

| Watch Class | %(n)^a^ |
| --- | --- |
| Azithromycin (IV) | 43.6%(17/39) |
| Azithromycin (oral) | 7.7%(4/52) |
| Cefepime | 68.4%(26/38) |
| Cefotaxime | 28.9%(13/45) |
| Ceftazidime | 50.9%(27/53) |
| Ceftriaxone | 26.3%(15/57) |
| Cefuroxime | 19.0%(4/21) |
| Ciprofloxacin (IV) | 61.7%(29/47) |
| Ciprofloxacin (oral) | 10.5%(6/57) |
| Clarithromycin (IV) | 39.1%(9/23) |
| Clarithromycin (oral) | 9.1%(5/55) |
| Erythromycin (oral) | 4.4%(2/45) |
| Ertapenem | 74.5%(38/51) |
| Fosfomycin (oral) | 43.5%(10/23) |
| Imipenem/Cilastatin | 78.2%(43/55) |
| Levofloxacin (IV) | 56.1%(23/41) |
| Levofloxacino (oral) | 23.1%(12/52) |
| Meropenem | 82.0%(41/50) |
| Moxifloxacin (oral) | 35.3%(12/34) |
| Moxifloxacin (IV) | 42.3%(11/26) |
| Piperacillin/Tazobactam | 70.4%(38/54) |
| Vancomycin (IV) | 76.4%(42/55) |
| Vancomycin (oral) | 50.0%(12/24) |

^a^ Values informed by hospitals; IV: intravenous

**Table S3**. Characteristics of the institutions and the score survey

| **Characteristics** | **n** | **Score, median (range)** |
| --- | --- | --- |
| **Complexity**  Medium  High | 15  42 | 54.7 (30.7-85.4)  72.6 (45-96.5) |
| **Bed number**  0-100  101-500  >500 | 11  36  10 | 57.4 (30.7-85.4)  69.2 (37-96.5)  74.7 (57.9-90.7) |

ASP: Antibiotic stewardship programs

| **File S1: Survey in Spanish** | | | | |
| --- | --- | --- | --- | --- |
| **Categoría** | **ENCUESTA PROA - 2019** | | | |
|  | **Pregunta** | **Respuesta** | **Asignación del puntaje** | **Puntos subcategoría** |
| **Organización del PROA** | 4. ¿Cuenta su establecimiento con un programa o grupo de trabajo que lidere el uso racional de los antimicrobianos? | Si | 14% del puntaje total, si la respuesta es "Si" | **20** |
|  |  | No |  |  |
|  | 5 ¿Cuántos años está funcionando este programa? Indique número de años: | - | No evaluable |  |
|  | 6. ¿Cuenta su establecimiento con una política institucional o un protocolo escrito de apoyo al programa que permita un uso racional de los antimicrobianos? ? | Si | 2% del puntaje total, si la respuesta es "Si" |  |
|  |  | No |  |  |
|  |  | No sé |  |  |
|  | 7. ¿Cuenta el equipo en que participa de un grupo o programa de uso racional de antimicrobianos con un documento institucional detallando las actividades y funciones del programa? | Si | 2% del puntaje total, si la respuesta es "Si" |  |
|  |  | No |  |  |
|  | 8. ¿Cuenta el equipo en que participa de un programa de uso racional de antimicrobianos con un documento institucional, propio que establece las funciones y la forma de coordinación de cada uno de sus integrantes? | Si | 2% del puntaje total, si la respuesta es "Si" |  |
|  |  | No |  |  |
| **Recurso humano** | 9.     Señale los profesionales desarrollan funciones de optimización de uso de antimicrobianos en su institución de salud (Marque todas las que apliquen): | Médico microbiólogo | 5% del puntaje total por cada profesional señalado: 1) 5% Médico microbiólogo; 2) 5% Infectólogo; 3) 5% Farmacéutico Clínico que desarrolle funciones de optimización antimicrobiana | **15** |
|  |  | Médico infectólogo o Internista capacitado |  |  |
|  |  | Cirujano |  |  |
|  |  | Médico paciente crítico |  |  |
|  |  | Farmacéutico Clínico capacitado |  |  |
|  |  | Farmacéutico asistencial capacitado |  |  |
|  |  | Enfermera |  |  |
|  |  | Tecnólogo Médico capacitado |  |  |
|  |  | Analista de datos/Especialista en tecnologías de la información |  |  |
|  |  | Soporte administrativo |  |  |
|  |  | Otro (señalar): |  |  |
|  | 10. ¿Cuántas horas semanales (exclusivas) le dedica cada uno de los integrantes señalados en la pregunta anterior al trabajo en el uso racional de los antimicrobianos | Médico microbiólogo | 1,67% del puntaje total, si cuenta con un profesional de cada grupo señalado 1) 1,67% Médico microbiólogo o Tecnólogo Médico ; 2) 1,67% Infectólogo; 3) 1,67% Farmacéutico Clínico, con **al menos 11 horas** dedicadas al uso racional de antimicrobianos. | **5** |
|  |  | Médico infectólogo |  |  |
|  |  | Cirujano |  |  |
|  |  | Médico paciente crítico |  |  |
|  |  | Farmacéutico Clínico capacitado |  |  |
|  |  | Farmacéutico asistencial capacitado |  |  |
|  |  | Enfermera |  |  |
|  |  | Tecnólogo Médico capacitado |  |  |
|  |  | Tecnólogo médico |  |  |
|  |  | Analista de datos/Especialista en tecnologías de la información |  |  |
|  |  | Soporte administrativo |  |  |
|  |  | Otro (señalar): |  |  |
|  | 11.   La implementación de un trabajo que controle el uso de los antimicrobianos en una institución sanitaria, requiere que sus integrantes sean especialistas en un conjunto de materias y habilidades que garanticen un desempeño competente en el programa. ¿Cuáles son los tópicos en los que se requiere capacitación para perfeccionar las competencias requeridas? Marque todas las que corresponda: | Tópicos de infectología | No evaluable | **0** |
|  |  | Pasantías o estadías de perfeccionamiento PROA |  |  |
|  |  | Capacitación en PROA |  |  |
|  |  | Cursos de ajuste de antibióticos a través de técnicas farmacocinética/farmacodinamia |  |  |
|  |  | Técnicas de toma de muestra de cultivos |  |  |
|  |  | Otros (Especifique) |  |  |
| **Actividades** | 12. ¿Qué actividades contempla la optimización del uso de los antimicrobianos en su institución? | Recomendación de cómo se debe hacer la toma de muestras microbiológicas | 5% del puntaje total, si se realiza 3 o más actividades listadas. Si realiza 2 actividades 3,333%, si realizan 1 actividad 1,667% y 0 actividad 0% | **5** |
|  |  | Autorización de uso de antimicrobianos restringidos |  |  |
|  |  | Recomendación sobre la duración de la terapia |  |  |
|  |  | Solicitar asesoría en casos de infecciones complejas o desconocidas |  |  |
|  |  | Revisión de la terapia a las 48-72 horas de su inicio |  |  |
|  |  | Recomendación de cambio de terapia endovenosa a oral |  |  |
|  |  | Suspensión automática de la terapia cuando la duración del tratamiento exceda el tiempo de tratamiento autorizado. |  |  |
|  |  | Asesoría en monitorización plasmática de antibióticos |  |  |
|  |  | Otros (Especifique) |  |  |
|  | 13. ¿Señale en que servicios clínicos del establecimiento, existe una supervisión del uso de los antimicrobianos? Marque todos los que correspondan y la frecuencia de visita todos los días, tres veces por semana, si existen solicitudes antimicrobianas restringidos, sólo con interconsulta | UCI | No evaluable | **0** |
|  |  | UTI |  |  |
|  |  | Medicina |  |  |
|  |  | Cirugía |  |  |
|  |  | Servicia de Urgencia Adulto |  |  |
|  |  | Servicio Urgencia Pediátrico |  |  |
|  |  | Pediatría |  |  |
|  |  | Neonatología |  |  |
|  |  | Otros, señale los 4 más relevantes no señalados anteriormente |  |  |
|  | 14. ¿Cómo realiza operativamente las actividades el equipo dedicado la supervisión del uso óptimo de los antimicrobianos? Marque todos los que correspondan | Visita directa al servicio | No evaluable | **0** |
|  |  | Visita directa al servicio según disponibilidad |  |  |
|  |  | Revisión directa en sala posterior a la prescripción del antimicrobiano |  |  |
|  |  | Revisión directa en sala previo a la dispensación del antimicrobiano |  |  |
|  |  | Revisión directa en sala previo a la dispensación del antimicrobiano con despacho autorizado hasta la evaluación del equipo PROA |  |  |
|  |  | Revisión de la solicitud de autorización documental |  |  |
|  |  | Sólo por interconsulta |  |  |
|  |  | Otra forma: Especifique |  |  |
|  |  | No se hace |  |  |
|  | 15. Respecto de las actividades que realiza la farmacia integrada al equipo que lidera el uso óptimo de los antimicrobianos , Marque todas las que apliquen: | La farmacia dispensa los antibióticos según procedimiento establecido por el PROA | 5% del puntaje total, si se realiza 3 o más actividades listadas. Si realiza 2 actividades 3,333%, si realizan 1 actividad 1,667% y 0 actividad 0% | **5** |
|  |  | Farmacia entrega en forma sistemática alertas al equipo PROA respecto de los tratamientos prolongados. |  |  |
|  |  | La farmacia entrega alertas al equipo PROA de los tratamientos que cumplen 3 a 5 días y son factibles de desescalar a vía oral. |  |  |
|  |  | La farmacia entrega alertas para la suspensión de profilaxis quirúrgica cuando exceden el tiempo establecido por el PROA |  |  |
|  |  | La farmacia entrega alertas al equipo PROA de los inicios de tratamientos con fármacos restringidos y de alto costo. |  |  |
|  |  | La farmacia realiza una estadística de los quiebres de stock por antibiótico |  |  |
|  |  | La farmacia entrega alertas de quiebre de stock |  |  |
|  |  | La farmacia avisa de errores de medicación sobre antimicrobianos |  |  |
|  |  | La farmacia avisa de solicitudes de ATB restringidos desde hospitales de la red |  |  |
|  |  | La farmacia avisa de solicitudes de despacho de ATB restringidos a pacientes que serán trasladados a la red |  |  |
|  |  | La farmacia cuenta con protocolos que garanticen administración segura de antimicrobianos |  |  |
|  |  | Utiliza para formulario especial para autorización de la prescripción de antimicrobianos |  |  |
|  |  |  |  |  |
|  | 16.Respecto de los antimicrobianos que requiere autorización para su uso, señale cuántos son restringidos y la cantidad total: | ¿Cuántos? (número): | 5 | **5** |
|  |  | ¿Cuáles? Dirigirse a lista de antimicrobianos en Anexo |  |  |
|  |  | Total de antimicrobianos en su hospital |  |  |
|  | 17. ¿Indique que profesionales visitan las salas revisando la respuesta de la terapia antibiótica para aquellos antimicrobianos de uso restringido? Marque todas las que correspondan. | Médico | No evaluable | **0** |
|  |  | Farmacéutico |  |  |
|  |  | Microbiólogo |  |  |
|  |  | Otro |  |  |
|  |  | Ninguno |  |  |
|  | 18.     En su establecimiento mencione ¿Qué profesionales sugieren ajustes de dosis en disfunción de algún órgano? Marque todas las que apliquen | Farmacéutico | No evaluable |  |
|  |  | Médico |  |  |
|  |  | Otro: Especifique |  |  |
|  |  | Ninguno |  |  |
|  | 19.Del equipo PROA, los profesionales señalados que sugieren ajustes de dosis para optimizar un tratamiento usando parámetros farmacocinéticos /farmacodinámicos (PK/PD), en casos de susceptibilidad reducida por parte del agente microbiano? Especifique todos los profesionales que lo realizan: | Farmacéutico | No evaluable |  |
|  |  | Médico |  |  |
|  |  | Microbiólogo |  |  |
|  |  | Otro: Especifique |  |  |
|  |  | Ninguno |  |  |
| **Guías de tratamiento** | 20. ¿Tiene su establecimiento implementadas guías de tratamiento para optimizar el uso de antibióticos de una o más de las siguientes infecciones? seleccione todas las que apliquen indicando si  corresponden a guías locales (del hospital ) o de otros organismos. | Neumonía adquirida en la comunidad | 10% del puntaje total si se cuenta con 3 o más guías de tratamiento de las infecciones nombradas anteriormente (independiente se es local o nacional/internacional). Si tiene 2 guías es un 6,66% y si tiene 1 guía es 3,33% | **10** |
|  |  | Infección del tracto urinario |  |  |
|  |  | Infección de piel y partes blandas |  |  |
|  |  | Profilaxis quirúrgica |  |  |
|  |  | Infección por S. aureus meticilino-resistente (SAMR) |  |  |
|  |  | Diarrea por C. Difficile |  |  |
|  |  | Influenza |  |  |
|  |  | Infecciones osteoarticulares |  |  |
|  |  | Infección intraabdominal |  |  |
|  |  | Meningitis |  |  |
|  |  | Infección del torrente sanguíneo |  |  |
|  |  | Neutropenia Febril |  |  |
|  |  | Infecciones en pacientes obstétricas |  |  |
|  |  | Infecciones en pacientes ginecológicas |  |  |
| **Vigilancia** | 21.En relación a vigilancia y monitoreo: Seleccione la(s) que correspondan. | Los datos de consumo de antibióticos se correlacionan con los datos de resistencia antibiótica | 10% del puntaje total, si tiene marcada la opción "Los datos de consumo de antibióticos se correlacionan con los datos de resistencia antibiótica se realizan datos de consumo de antibióticos correlacionados con datos de resistencia antibiótica" + Dosis Diaria Definida/100 días cama. (DDD) ó Días De Terapia/100 días cama (DOT) Si tiene solo "Los datos de consumo de antibióticos se correlacionan con los datos de resistencia antibiótica se realizan datos de consumo de antibióticos correlacionados con datos de resistencia antibiótica" son 5 puntos Si tiene marcada solo una opción entre "Dosis Diaria Definida/100 días cama. (DDD)" ó "Días De Terapia/100 días cama (DOT)" son 2,5- Si tiene ambas son 5 puntos | **10** |
|  |  | Dosis Diaria Definida/100 días cama. (DDD). |  |  |
|  |  | Días De Terapia/100 días cama (DOT) |  |  |
|  |  | Realiza evaluación de los costos asociados al consumo de antibióticos ($) |  |  |
|  |  | Realiza monitorización de algún indicador clínico: Especifique: |  |  |
|  |  | Realiza una evaluación del cumplimiento de las guías de tratamiento antimicrobiano por parte de los equipos tratantes |  |  |
|  |  | Realiza evaluación de la calidad de la prescripción de antimicrobianos |  |  |
|  | 22.En relación a las actividades que realiza el laboratorio de microbiología: Seleccione todas las que corresponda: | El informe microbiológico se entrega máximo en 48-72 horas | Solo se medirán 2: 1) El informe microbiológico se entrega máximo en 48-72 horas ; 2)Realiza al menos una vez al año medición de incidencia de agentes multirresistentes en muestras clínica.  Si tiene ambos es 10% si tiene el informe microbiológico en 48-72 horas Si tiene solo uno es 5% | **10** |
|  |  | El informe microbiológico se entrega sobre 72 horas |  |  |
|  |  | El laboratorio alerta de resultados críticos como hemocultivos positivos |  |  |
|  |  | Los informes de susceptibilidad se describen de manera estratificada |  |  |
|  |  | Realiza al menos una vez al año medición de incidencia de agentes multirresistentes en muestras clínicas |  |  |
|  |  | Los informes de resistencia se realizan en base a puntos de corte epidemiológicos |  |  |
|  |  | Los informes de resistencia se realizan siguiendo normas estandarizadas establecidas por el CLSI |  |  |
|  |  | Cuenta con técnicas rápidas para identificación de microorganismos resistentes |  |  |
| **Análisis y retroalimentación** | 23. ¿El PROA comparte o socializa mediante reportes u otros medios la información analizada, de los consumos de antimicrobianos, a los prescriptores de antimicrobianos? Seleccione todas las que corresponda | Anual | 5% del puntaje total, si se realiza de forma anual o semestral | **5** |
|  |  | Semestral |  |  |
|  |  | No se hace |  |  |
|  |  | Si, pero sólo con datos de algunos servicios |  |  |
|  |  | Ocasionalmente |  |  |
|  |  | Otro: Especifique |  |  |
|  | 24.En su establecimiento, se distribuye en forma periódica un informe acumulado de susceptibilidad a quienes prescriben? Marque todas las que correspondan. | Anual | 5% del puntaje total, si se realiza de forma anual o semestral | **5** |
|  |  | Semestral |  |  |
|  |  | No se hace |  |  |
|  |  | Otro: Especifique |  |  |
|  |  | Ocasionalmente |  |  |
|  | 25.Cómo se realiza la retroalimentación a quienes prescriben, acerca de cómo mejorar la prescripción de antibióticos? | Diario en la visita | No evaluable | **0** |
|  |  | En respuesta a la interconsulta |  |  |
|  |  | Semanalmente se presentan casos en una reunión |  |  |
|  |  | Otra (señalar). |  |  |
|  | 26.Como se educa al personal de salud para el uso adecuado de antibióticos? | En inducción presencial | 5% del puntaje total, si realiza alguna estrategia de educación (cualquiera excepto "no educa", "desconozco" y "otro") | **5** |
|  |  | En inducción por E-learning |  |  |
|  |  | En inducción - información escrita entregada |  |  |
|  |  | Actualización anual obligatoria |  |  |
|  |  | Actualización anual cada 2 años |  |  |
|  |  | Cursos con evaluación |  |  |
|  |  | Cursos sin evaluación |  |  |
|  |  | Reuniones clínicas |  |  |
|  |  | No educa |  |  |
|  |  | Desconozco |  |  |
|  |  | Otro |  |  |
|  |  |  | **Puntaje Total** | **100** |
|  |  |  |  |  |
|  | **ANEXO ANTIMICROBIANOS** | | | |
|  | Para todos los antimicrobianos que tienen respuesta es posible encontrar las siguientes situaciones: Arsenal y requiere autorización, Requiere autorización, Autorización y uso ocasional, Solo Arsenal Y Uso ocasional. Se construirá el siguiente indicador para todos los medicamentos con respuesta: N° Arsenal y requiere autorización*100 ----------------------------------------------- (N° Arsenal y requiere autorización+ Solo Arsenal ) Se saca regla de 3 para obtener el puntaje total: Si el indicador es de 100 se le da 5 puntos (máximo puntaje) | | | |
|  | ACICLOVIR AM, AMIKACINA SULFATO FA, AMOXICILINA CM, AMOXICILINA/ÁCIDO CLAVULÁNICO CM, AMOXICILINA-SULBACTAM 1000 MG/500 MG FA, AMPICILINA 500 mg FA, AMPICILINA/SULBACTAM FA 1,5 g ANFOTERICINA B DEOXICOLATO 50 mg FA, ANFOTERICINA LIPOSOMAL 50 mg FA, ANIDULAFUNGINA 100 mg FA, AZITROMICINA MG CM, AZITROMICINA mg FA, AZTREONAM EV, CASPOFUNGINA FA, CEFADROXILO VO, CEFAZOLINA 1 g FA, CEFEPIME FA, CEFOTAXIMA 1 g FA, CEFTAROLINA EV, CEFTAZIDIMA EV, Ceftazidima/Avibactam FA EV, Ceftolozano/Tazobactam FA EV, CEFTRIAXONE 1 g FA, CEFUROXIMA SODICA FA, CIPROFLOXACINO 200 mg FA 200 mg/100 Ml, CIPROFLOXACINO 500 mg CM, CLARITROMICINA 500 mg CM, CLARITROMICINA 500 mg FA, CLINDAMICINA AM, CLOXACILINA 500 mg FA, COLISTIN, COTRIMOXAZOL CM, COTRIMOXAZOL 400/80 FA, DAPSONA VO, DAPTOMICINA FA, DOXICICLINA VO, ERITROMICINA VO, ERTAPENEM 1 g FA, FLUCONAZOL 100 mg CP, FLUCONAZOL 2 mg/100 ml FA, FOSFOMICINA VO, FOSFOMICINA EV, GANCICLOVIR 500 mg FA, GENTAMICINA AM , IMIPENEM 500 mg FA, LEVOFLOXACINO CM, LEVOFLOXACINO FA, LINEZOLID 600 /300 ml, LINEZOLID VO, MACRODANTINA VO, MEROPENEM FA, METRONIDAZOL 500 mg/100 ml, MOXIFLOXACINA 400 mg CM, MOXIFLOXACINA 400 mg EV, NITROFURANTOINA VO, PEN. 1 MILLON FA, PEN.BENZATINA 1.200.00 FA, PIPERACILINA/TAZOBACTAM 4 g/ 0,5 g, TIGECICLINA FA, VANCOMICINA FA, VANCOMICINA VO, VORICONAZOL FA 200 CM, VORICONAZOL FA 200 mg | | | |
|  |  |  |  |  |
|  |  |  |  |  |
|  | FA: Frasco ampolla, AM: Ampolla, CM: Comprimido, CP: Cápsula | | | |
